# Effects of neuromuscular blocking agents on the clinical performance of i-gel^®^ and surgical condition in elderly patients undergoing hand surgery: a prospective randomized controlled trial

**DOI:** 10.7150/ijms.57489

**Published:** 2021-04-16

**Authors:** Choon-Kyu Cho, Minhye Change, Seok-Jin Lee, Tae-Yun Sung

**Affiliations:** Department of Anaesthesiology and Pain medicine, Konyang University Hospital, Myunggok Medical Research Center, Konyang University College of Medicine, Daejeon, Korea.

**Keywords:** neuromuscular blocking agents, surgery, aged, rocuronium

## Abstract

**Background:** The effects of neuromuscular blocking agents on the clinical performance of supraglottic airway devices and surgical condition in elderly patients undergoing hand surgery have not been established. We evaluated the effects of rocuronium on the clinical performance of an i-gel^®^ supraglottic device and surgical condition in elderly patients undergoing orthopedic hand surgery.

**Methods:** Patients aged 65-85 years were randomized to receive either rocuronium (rocuronium group) or saline (control group). We compared the rates of successful insertion of the i-gel on the first attempt as a primary outcome and also assessed the adequacy of i-gel maintenance during controlled ventilation, anesthetic requirement, surgical condition, and recovery time.

**Results:** The rates of successful insertion of the i-gel on a first attempt were 93.1% in the rocuronium group versus 82.1% in the control group (*P* = 0.423). Peak inspiratory pressure (PIP) was lower in the rocuronium group than in the control group (15.2 vs. 17.9 cmH_2_O, respectively, *P* = 0.028). Spontaneous breathing was less common in the rocuronium group (24.1% vs. 57.1%, respectively, *P* = 0.011). The requirement of additional fentanyl to suppress spontaneous breathing or patient movement was less in the rocuronium group than in the control group (24.1% vs. 50.0%, respectively, *P* = 0.043). Surgical condition did not differ between the two groups. Recovery time was shorter in the rocuronium group than in the control group (8.4 vs. 9.9 min, respectively, *P* = 0.030).

**Conclusions:** Rocuronium did not enhance the success rate of inserting the i-gel^®^ or the surgical condition in elderly patients. However, using rocuronium reduced PIP, the frequency of spontaneous breathing, the requirement for additional fentanyl and patients' recovery time.

## Introduction

Neuromuscular blocking agents (NMBAs), drugs that interrupt neuromuscular transmission at neuromuscular junctions via an acetylcholine receptor blockade, are used to paralyze skeletal muscles 1. The use of NMBAs during general anesthesia has the advantages of facilitating endotracheal intubation during induction of anesthesia, providing sufficient surgical muscle relaxation, assisting with controlled ventilation, and reducing the anesthetic requirement during surgery 2,3. However, NMBAs are the most common cause of perioperative anaphylaxis 4, and their use is a risk factor for accidental intraoperative awareness 5. Furthermore, using intraoperative NMBAs may result in postoperative residual neuromuscular block, which can increase postoperative pulmonary complications 6. Although there have been conflicting results regarding the association between age and postoperative residual neuromuscular block 7-9, some studies have found that the incidence of postoperative residual neuromuscular block increases with age 8,9, occurring more frequently in older adults than in younger patients 6.

Advances in supraglottic airway devices (SADs) have reduced the need for NMBAs during general anesthesia as an alternative to conventional airway management using an endotracheal tube 2. However, the need for NMBAs when inserting and maintaining SADs is controversial, and studies have reported varying results depending on the proficiency of the person who inserted the SAD, the type of surgery, the kind of SAD, and the characteristics of the patient, such as obesity and age 10-13. Therefore, to avoid adverse events related to the use of NMBAs, their use in patients undergoing surgery under general anesthesia using SADs should be individualized and confined to surgeries where it is mandatory 11,14.

The pharmacokinetics and pharmacodynamics of NMBAs may change in the elderly due to age-related changes in physiology 3. In addition, the clinical efficacy of SADs is not the same as that in younger patients due to structural changes in the pharyngeal airway with age 15. Nevertheless, few studies have determined the effects of NMBAs on the clinical performance of SADs in elderly patients 16. Moreover, it is unclear whether not using an NMBA affects the surgical condition of elderly patients undergoing hand surgery with SADs 10,17. Therefore, we designed this study to evaluate the effects of an NMBA (rocuronium) on the clinical performance of the i-gel^®^ supraglottic device (i-gel) and surgical condition in elderly patients undergoing orthopedic hand surgery.

## Methods

This prospective, randomized, controlled study was approved by the Konyang University Hospital Institutional Review Board (KYUH 2018-01-001-001) and was registered at the Korea Clinical Research Information Service (https://cris.nih.go.kr/, permit number: KCT 0003616). Written informed consent was obtained from the patients and/or from a legal representative. This study adhered to Consolidated Standards of Reporting Trials (CONSORT) guidelines.

Patients aged 65-85 years old of American Society of Anesthesiologist Physical Status classification I-III and undergoing elective orthopedic hand surgery with an expected surgical duration < 2 h were included in the study. The exclusion criteria were morbid obesity (body mass index > 35 kg/m^2^), pharyngeal pathology, gastroesophageal reflux disease, a recent history of upper respiratory infection, loose teeth, psychiatric disease, or cognitive disorder.

Patients were randomly allocated to the rocuronium group or the control group in a 1:1 ratio using online randomization software (Researcher Randomizer; www.randomizer.org). Immediately before anesthesia was induced, a trained researcher who prepared the study drugs (rocuronium or normal saline) opened an opaque envelope containing the group allocations. The trained researcher was not involved in collecting the study data or inpatient care. Intra- and postoperative anesthetic management, assessments of outcome variables, and surgery in this study were conducted by two anesthesiologists and one surgeon, who were blinded to the patient allocation.

All patients fasted for at least 8 hours and entered the operating room without premedication. Before preparation for routine monitoring, each patient's dental status and modified Mallampati classification (class 1 = soft palate, fauces, uvula, and pillars visible; 2 = soft palate, fauces, uvula visible; 3 = soft palate, base of uvula visible; 4 = none of the soft palate visible) 18 were assessed by the anesthesiologist. Thereafter, pulse oximetry, electrocardiography, noninvasive blood pressure, and bispectral index (BIS) monitoring were applied. Patients were preoxygenated with 100% O_2_ at 8 L/min fresh gas flow for at least 3 min. Anesthesia was induced with 1 mg/kg lidocaine, 1-2 mg/kg propofol, and 1 μg/kg fentanyl. After confirming loss of consciousness and loss of the eyelid reflex, facemask ventilation was initiated with 3%-5% sevoflurane. The rocuronium group received 0.6 mg/kg intravenous rocuronium, while the control group received the same volume of normal saline. To ensure sufficient neuromuscular blockade in the rocuronium group 19 and adequate depth of anesthesia in the control group, there was a pause of at least 2 min after injecting the rocuronium or normal saline. The i-gel (Intersurgical Ltd., Wokingham, Berkshire, UK) was inserted if the BIS was < 60 in either group. The i-gel was inserted in both groups by the same anesthesiologist, who had clinical experience of more than 200 insertions of the i-gel.

The size of the i-gel was chosen based on the patient's body weight according to the manufacturer's instructions 20. The posterior and lateral sides of the device were lubricated with water-soluble gel before the first insertion attempt. The primary outcome of this study was the rate of successful insertion of the i-gel on the first attempt. The i-gel was considered successfully inserted when normal square-wave capnography traces appeared, there was no audible leakage from the mouth of the patient at a peak inspiratory pressure (PIP) ≤ 20 cmH_2_O, and bilateral chest wall movements were observed 21. Up to three insertion attempts were allowed, and each trial allowed manipulations for proper placement of the i-gel, such as neck extension or flexion, adjustment of the inserted depth by pushing or pulling on the device, and jaw thrust. If the third insertion attempt was unsuccessful, it was recorded as a failed i-gel insertion. In the case of a failure, endotracheal intubation was performed and the case was excluded from further evaluation. The ease of insertion was evaluated using a visual analog scale (VAS, 0 = extremely easy, 100 = extremely difficult) by the anesthesiologist who attempted the insertion 13. Insertion time, which was defined as the time from picking up the device to successful insertion, was measured by the other anesthesiologist.

After successful insertion of the device, volume-controlled mechanical ventilation was started at a tidal volume of 7 mL/kg and a respiratory rate of 12 breaths/min. Then, the respiratory rate was adjusted to maintain end-tidal CO_2_ at 35-40 mmHg. The position of the i-gel was assessed using fiberoptic bronchoscopy and graded with the Brimacombe score (grade 1 = vocal cords not visible; 2 = vocal cords and anterior epiglottis visible; 3 = vocal cords and posterior epiglottis visible; 4 = only vocal cords visible) 22. Oropharyngeal leak pressure (OLP) and gastric insufflation were evaluated after grading the fiberoptic view. OLP was measured by detecting an audible leak over the patient's mouth upon closing the expiratory valve of the anesthesia breathing system to 40 cmH_2_O at a fixed fresh gas flow of 3 L/min 21. The presence of a sound in the epigastrium via stethoscope was considered gastric insufflation when the OLP was measured 21.

Anesthesia was maintained during surgery with 1%-3% sevoflurane in 50% nitrous oxide. The concentration of end-tidal sevoflurane was adjusted to maintain a BIS of 40-60, and the minimum end-tidal sevoflurane concentration to maintain the BIS < 60 was recorded. PIP was measured in the absence of spontaneous breathing or patient movement, and the highest PIP during surgery was recorded. Intraoperative spontaneous breathing and patient movement were recorded, and both were treated with an increase in sevoflurane concentration and/or a bolus injection of additional fentanyl (0.5-1 μg/kg) at the discretion of the anesthesiologist. Nevertheless, additional rocuronium (5-10 mg) was administered if these problems were not resolved or the surgeon required additional neuromuscular block.

After completing the surgery, surgical condition scores using a four-point scale (1 = poor, surgery cannot proceed because of muscle contractions or gross patient movement; 2 = acceptable, muscle contractions causing some interference in the surgical procedure; 3 = good, only a few muscle contractions that do not affect the surgical procedure; and 4 = excellent, no muscle contractions.) 10 were assessed by a single (board-certified) surgeon who performed the surgery. Sevoflurane and nitrous oxide were stopped and manual ventilation with 100% O_2_ at 6 L/min fresh gas was commenced. To reverse the neuromuscular blockade, the rocuronium group received 40 μg/kg neostigmine and 10 μg/kg glycopyrrolate, while the control group received the same volume of saline. The i-gel was removed after confirming that the patient obeyed verbal commands (e.g., “open your eyes”), spontaneously respired (respiratory rate 10-20 breaths/min), and the BIS was ≥80. The time from turning off the sevoflurane to removing the i-gel was defined as recovery time and was recorded. During emergence and removal of the i-gel, any adverse events, such as desaturation (peripheral oxygen saturation < 95%), coughing, bronchospasm, tongue/lip/teeth injury, or a blood-tinged tip of the i-gel were recorded. Postoperative pain and sore throat were evaluated in the post-anesthetic care unit (PACU) using a numerical rating scale (NRS) score (0 = no pain/sore throat, 10 = worst pain/sore throat imaginable). If the patient asked for an analgesic due to pain or the anesthesiologist judged that analgesic administration was necessary based on the NRS score, 0.5-1 μg/kg fentanyl was administered. If the patient complained of nausea or vomiting, 10 mg metoclopramide was given.

### Statistical analyses

In previous studies of adult patients, the differences in the rate of successful insertion of the i-gel and LMA-ProSeal^®^ on the first attempt between control and rocuronium groups were reported as 37% and 25%, respectively 12,13. In addition, the rate of successful insertion of i-gel on the first attempt with rocuronium was reported as 94.7% in elderly patients 21. Based on these previous reports 12,13,21, to detect a 30% difference in this rate between control and rocuronium groups with a power of 0.8 and a two-sided α-value of 0.05, 27 patients were required per group. Considering a dropout rate of 5%, 29 patients per group were recruited.

The statistical analyses were performed using PASW Statistics version 18 (IBM SPSS Inc., Chicago, IL, USA). After assessing the data distribution using the Kolmogorov-Smirnov test, continuous variables were analyzed using the Student's *t*-test or the Mann-Whitney *U*-test and are presented as mean ± standard deviation or median (interquartile range) values. Categorical variables were analyzed using the χ^2^ test, χ^2^ test for trends (linear-by-linear association), or Fisher's exact test, where appropriate, and are presented as a number or percentage (%). A two-sided *P* < 0.05 was considered significant.

## Results

**Figure 1:**
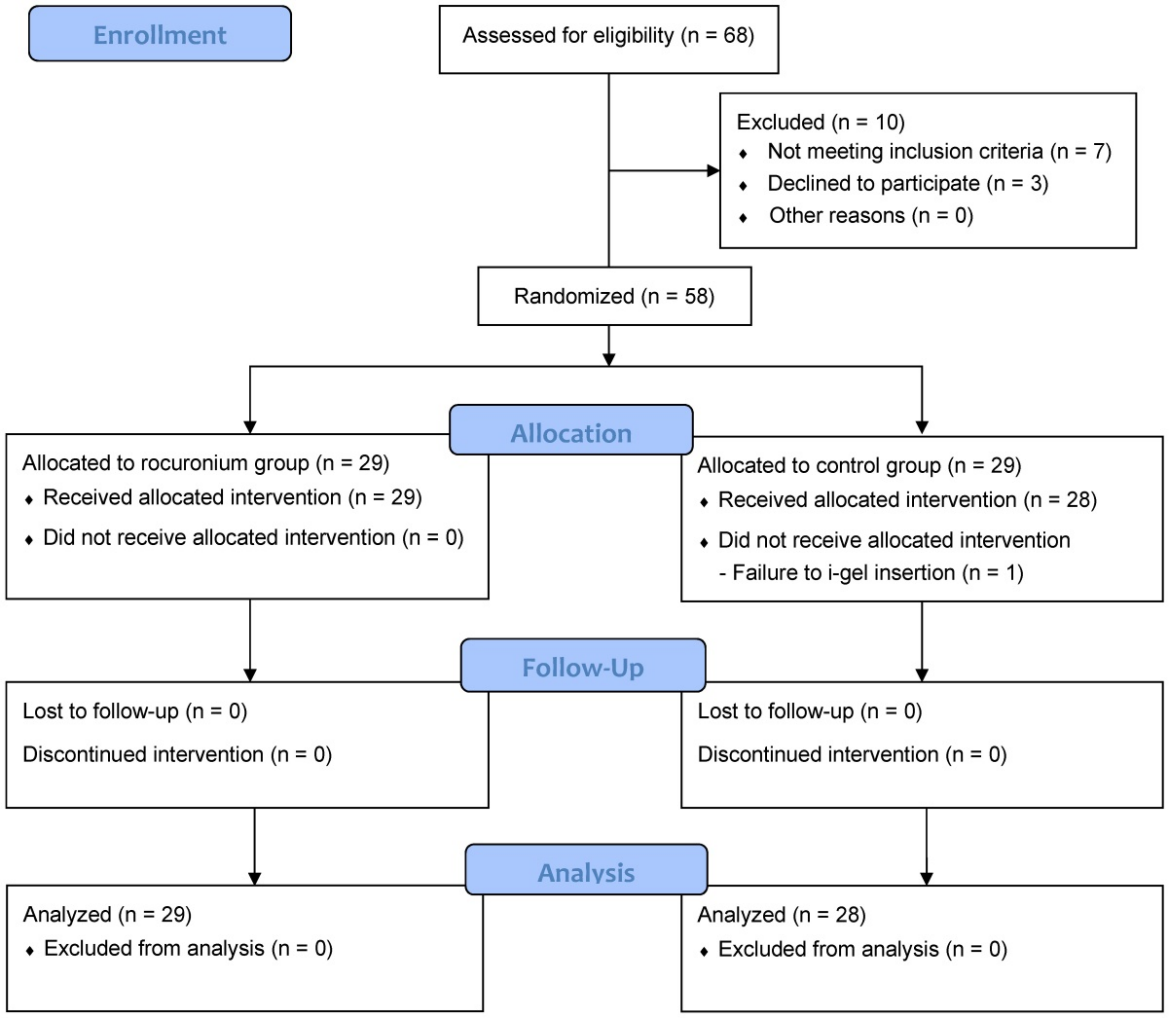
Flow diagram of patient enrollment.

A total of 68 patients were assessed for eligibility, and 10 were excluded; thus, 58 were randomly allocated either to the rocuronium group or the control group. Thereafter, there was one insertion failure in the control group. Consequently, outcome variables measured during maintenance of anesthesia and recovery were analyzed in the remaining 29 patients in the rocuronium group and 28 patients in the control group (Fig. 1).

The patient characteristics of the two groups were similar (Table 1).

The outcome variables related to inserting the i-gel are presented in Table 2. Although the rates of successful insertion on the first attempt showed a mean difference of 10.3%, the difference was not significant (93.1% in the rocuronium group vs. 82.8% in the control group, 95% confidence interval [CI]: 7.5% to 28.3%, *P* = 0.423). The numbers of attempts to achieve successful insertion, insertion times, and ease of insertion were also not significantly different between the groups.

The outcome variables measured during maintenance of anesthesia are shown in Table 3. PIP was significantly lower in the rocuronium group than in the control group (15.2 vs. 17.9 cmH_2_O, respectively; mean difference -2.65; 95% CI: 5.0-0.3; effect size 0.604; *P* = 0.028). The frequency of spontaneous breathing was significantly lower in the rocuronium group than in the control group (24.1% [7/29] vs. 57.1% [16/28], respectively; mean difference -33.0%; 95% CI: 53.2-7.5%, *P* = 0.011), but intraoperative patient movement did not differ between the two groups. Fewer patients in the rocuronium group required additional fentanyl during the surgery than in the control group (24.1% [7/29] vs. 50.0% [14/28], respectively; mean difference -25.9%; 95% CI: 46.9-0.9%; *P* = 0.043). OLP, gastric insufflation, fiberoptic view, patients requiring additional rocuronium, and the minimum end-tidal sevoflurane concentrations to keep the BIS < 60 were not significantly different between the two groups.

The surgical condition scores and recovery data are shown in Table 4. The surgical condition scores were rated as excellent in all patients in the rocuronium group, while 92.9% (26/28) of patients were excellent, one was good, and the other was poor in the control group (*P* = 0.196). Recovery time was significantly shorter in the rocuronium group than in the control group (8.4 vs. 9.9 min, respectively; mean difference -1.41; 95% CI for the difference -2.68 to -0.14; effect size 0.627; *P* = 0.030). Adverse events during emergence, NRS for pain/sore throat, and the number of patients requiring a postoperative analgesic or antiemetic in the PACU did not differ between the groups.

## Discussion

This study demonstrated that an NMBA had no significant effect on inserting the i-gel in elderly patients, as assessed by the rate of successful insertion on the first attempt, number of attempts for successful insertion, insertion time, and ease of insertion. In addition, although an NMBA was not administered, 96.4% (27/28) of the elderly patients who underwent orthopedic hand surgery showed good or excellent surgical condition in the control group. However, patients who did not receive the NMBA had a higher PIP, more frequent spontaneous breathing during mechanical ventilation, and an increased requirement for additional fentanyl to suppress spontaneous breathing or movement. Recovery time was delayed in patients who did not receive the NMBA.

Augmented fitting of a SAD in the oral cavity by sufficient relaxation of the pharyngeal muscles and increased upper airway collapsibility has been suggested as an explanation for the increased efficacy of SADs when NMBAs were administered compared to when NMBAs were not used in adult patients 12,13. However, as age increases, the sensitivity of upper-airway reflexes to stimuli and genioglossus negative pressure reflex, the main mechanism for maintaining pharyngeal patency, decreases, thus upper-airway collapsibility increases in the elderly 23,24. For these reasons, the facilitating effects of NMBAs on the insertion of SADs may be attenuated in elderly patients. This assumption is supported by the results of this study, indicating that rocuronium did not significantly improve any of the outcome variables associated with inserting the i-gel.

The rates of successful insertion of the i-gel on the first attempt were 94.7% and 94.3% in previous studies performed in paralyzed elderly patients 20,21, which are comparable to the 93.1% in the rocuronium group of this study. In contrast, these rates were 86% and 93.3% in non-paralyzed adult patients 25,26, a slightly higher trend than the 82.8% of the control group in our study. However, considering that a longer insertion time and a lower success rate are achieved in elderly patients than younger adult patients due to structural changes in the pharyngeal airway that occur with aging 15, the results of our study are understandable.

Only a few studies have compared the effects of an NMBA on SAD sealing pressure 11-13, but the results have been inconsistent; administration of an NMBA increased the sealing pressure of the i-gel 12 and the LMA-ProSeal^®^ 13 in adult patients, while another study found that an NMBA did not change the airway sealing pressure of the LMA-ProSeal^®^ in adult female patients 11. Nevertheless, none of these studies evaluated the grade of fiberoptic view. In contrast, the grade of fiberoptic view was evaluated in the present study and did not show a group difference. This means that the anatomical position in which the tip of the i-gel was placed was similar in the two groups. Thus, OLP was comparable between the two groups in our study.

During mechanical ventilation, advantages of NMBAs include increased chest-wall compliance, suppressed respiratory movements, prevention of patient-ventilator dyssynchrony, and reduced peak inspiratory and plateau pressures 27. Similarly, PIP and the number of patients who exhibited spontaneous breathing were significantly lower in the rocuronium group than in the control group. In addition, intraoperative patient movement was not observed in any patient in the rocuronium group. As PIP increases, the risk for gastroesophageal insufflation also increases, which may increase the risk for pulmonary aspiration 28. Although no significant difference was observed in our study, the rate of gastric insufflation was nearly three times less in the rocuronium group than in the control group (10.3% vs. 32.1%, respectively, *P* = 0.056). Therefore, an NMBA should be considered when PIP is expected to increase during mechanical ventilation via an i-gel, such as in cases of obesity, laparoscopic surgery, head-down position, or restrictive lung disease 29.

Although NMBAs are commonly used to improve the surgical condition 2,3, the effects of NMBAs on surgical condition are diverse, depending on the depth of the neuromuscular block (i.e., deep vs. moderate), type of surgery (e.g., laparoscopic vs. open surgery), age of the patient (e.g., children vs. adults), and airway management device (e.g., endotracheal tube vs. SAD) 10,11,30. Therefore, it may be necessary to use NMBAs prudently depending on the characteristics of the surgery and of the patient when SADs are applied for airway management. In this study, patients' surgical condition was comparable regardless of rocuronium administration. However, this result should be interpreted cautiously. This may not mean that NMBAs are unnecessary to improve surgical condition, because all patients in the rocuronium group were rated excellent for surgical condition.

Another remarkable result is that recovery times were shorter in patients who received rocuronium. This is inconsistent with previous findings, as not using an NMBA shortened recovery in children undergoing inguinal herniorrhaphy 10 and in adult women undergoing laparoscopic gynecological surgery 11. The main reason for this discrepancy appears to be because additional intraoperative fentanyl administration was more frequent in patients who did not receive rocuronium in our study than in a previous study 10. Fentanyl enhances the sedative effects of inhalation anesthetics, such as sevoflurane 31. Furthermore, the duration of action of fentanyl is prolonged in the elderly, and fentanyl itself is a cause of delayed recovery 32.

This study had several limitations. First, because the sample size calculation was performed only for the rate of successful insertion on the first attempt, which was the primary outcome, this study may have been underpowered to assess the remaining outcome variables. Second, as all i-gels were inserted by a single experienced anesthesiologist, the findings of this study, particularly the outcome variables related to inserting the i-gel, may not extend to inexperienced users. The rate of successful insertion of an i-gel on the first attempt is only 11.4% without rocuronium and 48.6% with rocuronium when novice personnel insert the i-gel in adult patients 12, which is considerably lower than the rate in this study. Third, in this study, the surgical condition was not measured using an objective evaluation tool 33, but was evaluated subjectively based on the opinion of a single board-certified surgeon. Therefore, depending on the evaluator, the results for the surgical condition may not be consistent. Finally, although sevoflurane was adjusted by the same target BIS value (40-60) in both groups and minimum end-tidal sevoflurane concentrations were adjusted to keep the BIS < 60, which was comparable in the two groups, it was difficult to determine the exact dose of sevoflurane administered during anesthesia. Sevoflurane is associated with dose-dependent suppression of minute ventilation and neuromuscular function 34, which may have affected the results of this study, such as spontaneous breathing, surgical condition, and recovery time. Therefore, future studies require the application of total intravenous anesthesia to rule out the effects of inhalational anesthetics on neuromuscular blockade and to facilitate a comparison of the anesthetic dose administered.

In conclusion, rocuronium did not improve the i-gel insertion success rate or the surgical condition in elderly patients undergoing orthopedic hand surgery. However, anesthesia using the i-gel without rocuronium led to increased PIP and frequency of spontaneous breathing during controlled mechanical ventilation, which may require anesthesiologists to manage the ventilator more carefully intraoperatively. In addition, increases in intraoperative requirements of anesthetics such as fentanyl may lead to delayed recovery. Our findings imply that rocuronium should be administered discreetly to elderly patients undergoing orthopedic hand surgery under general anesthesia using an i-gel to reduce ventilator problems or anesthetics requirements, rather than be routinely administered.

## Figures and Tables

**Table 1:** Patient characteristics

	Rocuronium (*n* = 29)	Control (*n* = 29)	*P*
Age, year	71.6 ± 5.1	70.5 ± 4.2	0.299
Sex, male/female	6/23	8/21	0.698
Body mass index, kg/m^2^	24.5 ± 3.3	23.5 ± 5.3	0.334
ASA classification, I/II/III	1/23/3	3/22/4	0.775
Modified Mallampati classification, 1/2/3/4	1/10/17/1	3/13/13/0	0.086
Dental status, total/partial/edentulous	21/6/2	19/8/2	0.769
Duration of surgery, min	51.1 ± 23.1	44.5 ± 18.7	0.235
Duration of anesthesia, min	69.1 ± 24.5	62.1 ± 19.1	0.230
i-gel size, 3/4	22/7	20/9	0.704

Values are mean ± standard deviation or numbers; ASA: American Society of Anesthesiologists.

**Table 2:** Comparison of outcome variables related to inserting the i-gel

	Rocuronium (*n* = 29)	Control (*n* = 29)	*P*
Successful insertion at first attempt	27 (93.1%)	24 (82.8%)	0.423^†^
Number of attempts for successful insertion (1/2/3/fail)	27/1/1/0	24/3/1/1	0.254^‡^
Insertion time, *s*	17.0 (14.0-20.5)	20.0 (16.3-24.8)^*^	0.069^§^
Ease of insertion, VAS	20.0 (10.0-30.0)	25.0 (15.0-33.8)	0.148^‡^

Values are numbers (%), numbers, or the median (interquartile range); VAS: visual analog scale (0 = extremely easy, 100 = extremely difficult). ^*^n = 28. ^†^Fisher's exact test, ^‡^χ^2^ test for trends (linear-by-linear association), ^§^Mann-Whitney *U*-test.

**Table 3:** Comparison of outcome variables measured during maintenance of anesthesia

	Rocuronium (*n* = 29)	Control (*n* = 28)	*P*
Fiberoptic view, 1/2/3/4	1/17/11/0	1/18/7/2	0.939^*^
Oropharyngeal leak pressure, cmH_2_O	22.2 ± 4.4	22.6 ± 2.6	0.677^†^
Gastric insufflation	3 (10.3%)	9 (32.1%)	0.056^‡^
Peak inspiratory pressure, cmH_2_O	15.2 ± 4.0	17.9 ± 4.9	0.028^†^
Spontaneous breathing	7 (24.1%)	16 (57.1%)	0.011^§^
Patient movement	0 (0%)	3 (10.7%)	0.112^‡^
Sevoflurane, vol%	2 (1.8-2.2)	2 (2-2.3)	0.427^ ΙΙ^
Additional fentanyl	7 (24.1%)	14 (50.0%)	0.043^§^
Additional rocuronium	0 (0%)	0 (0%)	NA

Values are mean ± standard deviation, numbers (%), number, or median (interquartile range). Fiberoptic view: 1 = vocal cords not visible; 2 = vocal cords and anterior epiglottis visible; 3 = vocal cords and posterior epiglottis visible; 4 = only vocal cords visible, Sevoflurane: minimum end-tidal sevoflurane concentrations to keep BIS < 60, NA: not applicable. ^*^χ^2^ test for trends (linear-by-linear association), ^†^Student's *t*-test, ^‡^Fisher's exact test, ^§^χ2 test, ^ΙΙ^Mann-Whitney *U*-test.

**Table 4:** Surgical condition scores and recovery data

	Rocuronium (*n* = 29)	Control (*n* = 28)	*P*
**In operating room**			
Surgical condition score			0.196^*^
1. Poor	0 (0%)	1 (3.6%)	
2. Acceptable	0 (0%)	0 (0%)	
3. Good	0 (0%)	1 (3.6%)	
4. Excellent	29 (100%)	26 (92.9%)	
Recovery time, min	8.4 ± 1.9	9.9 ± 2.8	0.030^†^
Adverse events during emergence			
Coughing	1 (3.4%)	1 (3.6%)	> 0.999^‡^
**In PACU**			
NRS for pain	3 (1.3-6.0)	3 (2.0-7.0)	0.248^§^
NRS for sore throat	0 (0-0.5)	0 (0-0)	0.545^§^
Analgesics (fentanyl)	9 (31.0%)	8 (28.6%)	0.839^ ΙΙ^
Antiemetics (metoclopramide)	1 (3.4%)	0 (0%)	> 0.999^‡^

Values are mean ± standard deviation, numbers (%), or median (interquartile range). Recovery time: Time from stopping sevoflurane administration to removal of the i-gel, PACU: post-anesthetic care unit, NRS: numerical rating scale (0 = no sense of pain/ sore throat, 10 = worst imaginable sense of pain/sore throat). ^*^χ^2^ test for trends (linear-by-linear association), ^†^Student's *t*-test, ^‡^Fisher's exact test, ^§^Mann-Whitney *U*-test, ^ΙΙ^χ^2^ test.
